# Evaluation of Grazing Behavior and Its Associations with Obesity, Emotion Regulation Difficulties, and Problematic Smartphone Use in Adolescents with Essential Hypertension

**DOI:** 10.3390/children13091233

**Published:** 2026-09-11

**Authors:** Gunes Isik, Cansu Mercan Isik, Masum Ozturk

**Affiliations:** 1 Pediatric Nephrology, Sivas Cumhuriyet University, 58140 Sivas, Türkiye; 2 Child and Adolescent Psychiatry, Sivas Cumhuriyet University, 58140 Sivas, Türkiye; cansumercan@cumhuriyet.edu.tr; 3 Child and Adolescent Psychiatry, Dicle University, 21280 Diyarbakir, Türkiye; masum.ozturk@dicle.edu.tr

**Keywords:** grazing behavior, essential hypertension, obesity, emotion regulation difficulties, problematic smartphone use, hepatic steatosis, adolescents

## Abstract

**Highlights:**

**What are the main findings?**

- A significant positive correlation was found between total Grazing Questionnaire scores and BMI (r = 0.252; *p* = 0.006), emotion regulation difficulties (r = 0.187; *p* = 0.041), and problematic smartphone use (r = 0.315; *p* < 0.001).
- A statistically significant difference was observed between the essential HTN and control groups in total Grazing Questionnaire scores [F(1, 117) = 4.85, *p* = 0.030, η^2^p = 0.040].

**What are the implications of the main findings?**

- Grazing behavior was positively associated with higher BMI, emotion regulation difficulties, and problematic smartphone use in adolescents.
- Incorporating assessment of grazing and related eating behaviors into the evaluation of adolescents with essential hypertension may help identify those who could benefit from comprehensive lifestyle and behavioral support.

**Abstract:**

**Background:** This study aimed to compare grazing behavior scores between adolescents with essential hypertension (HTN) and controls. It also investigated the associations of grazing behavior with obesity, emotion regulation difficulties (ERD), problematic smartphone use, and screen time. **Methods:** This cross-sectional case–control study enrolled 60 adolescents with essential HTN and 60 controls selected via frequency matching based on age and sex, all aged 12–18 years. The diagnosis of essential HTN was confirmed using 24-h ambulatory blood pressure monitoring (ABPM). Grazing behavior, ERD, problematic smartphone use, screen time and body mass index (BMI) were assessed in all participants. Group comparisons and associations between grazing behavior and the study variables were analyzed. **Results:** The essential HTN and control groups were comparable with respect to age and sex distribution (*p* > 0.05). The mean BMI in the essential HTN group was 31.18 ± 6.81 kg/m^2^, which was significantly higher than that in the control group (23.35 ± 4.47 kg/m^2^) (*p* < 0.001). A significant positive correlation was found between total Grazing Questionnaire (GQ) scores and BMI (r = 0.252; *p* = 0.006), ERD (r = 0.187; *p* = 0.041), and problematic smartphone use (r = 0.315; *p* < 0.001). A statistically significant difference was observed between the essential HTN and control groups in total GQ scores [F(1, 117) = 4.85, *p* = 0.030, η^2^p = 0.040]. After adjustment for BMI, participants with hepatic steatosis had significantly higher total GQ scores (*p* = 0.019, η^2^p = 0.138), Grazing Behaviors subscale scores (*p* = 0.046, η^2^p = 0.107), and Uncontrollability subscale scores (*p* = 0.025, η^2^p = 0.123) than those without hepatic steatosis. **Conclusions:** Adolescents with essential HTN had higher grazing behavior scores than controls after adjustment for BMI. Grazing behavior was also associated with ERD and problematic smartphone use across the study sample. The findings regarding grazing behavior and hepatic steatosis warrant cautious interpretation, as the subgroup analysis was exploratory and included a relatively small number of participants.

## 1. Introduction

Primary (essential) hypertension (HTN) is an increasingly important public health problem in adolescence, with potential consequences extending into adulthood. Although its prevalence varies according to country, race, and sex, childhood HTN has been reported in approximately 4–6.7% of children and adolescents in different studies [1,2,3]. Essential HTN is multifactorial, with obesity, sedentary lifestyle, unhealthy dietary habits, male sex, positive family history and stress among the major recognized risk factors [4]. In addition to these established cardiometabolic and lifestyle factors, increasing attention has been directed toward behavioral and psychological characteristics that may accompany obesity and HTN, including problematic eating behaviors, grazing, ERD, sedentary behavior, and problematic screen use [5,6,7].

Grazing is a problematic eating behavior characterized by the repeated and often unplanned consumption of relatively small to medium amounts of food throughout the day, irrespective of hunger or satiety [8]. This eating pattern may contribute to excessive overall energy intake and has been associated with unhealthy dietary behaviors and increased body weight [9,10]. Previous studies have also demonstrated associations between grazing, loss of control over eating, and higher BMI [11,12]. These findings are particularly relevant to pediatric HTN, as dietary patterns involving frequent intake of energy-dense foods and snacks may contribute to cardiometabolic risk. In addition, the nutritional composition of frequently consumed snacks, particularly their sugar and sodium content, may have implications for blood pressure and metabolic health [13,14]. Consistent with this relationship between dietary patterns and HTN, higher Dietary Inflammatory Index scores have been associated with childhood HTN, suggesting that dietary patterns may play an important role in pediatric blood pressure regulation [15]. Behavioral factors related to self-regulation may also be relevant to problematic eating behaviors.

The increasing use of digital devices and prolonged screen exposure have been associated with sedentary behavior and adverse cardiometabolic outcomes, including obesity and HTN [16,17]. Problematic internet use has been associated with impaired sleep and nutritional quality, as well as a higher prevalence of physical and mental health problems [18]. In addition, negative body image in overweight and obese adolescents has been associated with emotional eating [19], while obesity has been linked to mental health problems such as anxiety and depression, as well as psychosocial difficulties including low self-esteem, impaired social functioning, and weight-related stigma [20,21].

These findings suggest that psychological and behavioral factors may accompany obesity and problematic eating behaviors. In this context, problematic smartphone use and ERD may represent additional behavioral and psychological dimensions relevant to grazing behavior in adolescents with essential HTN. Despite increasing evidence linking dietary behaviors, obesity, psychological characteristics, and digital-device use with cardiometabolic health, limited information is available regarding grazing behavior in adolescents with essential HTN, particularly when hypertension is characterized using 24-h ambulatory blood pressure monitoring (ABPM). In particular, the extent to which grazing is associated with ERD and problematic smartphone use in this population remains insufficiently understood. Therefore, the present study sought to assess grazing behavior, ERD, and problematic smartphone use in adolescents with essential HTN by comparing them with a control group. Our goal is to raise awareness of grazing behavior among adolescents with essential HTN and to emphasize the importance of considering this behavior in comprehensive, multidisciplinary clinical management.

## 2. Materials and Methods

### 2.1. Study Design and Population

This cross-sectional, case–control comparative study included adolescents aged 12–18 years with essential HTN who presented to the Pediatric Nephrology Outpatient Clinic of Sivas Cumhuriyet University Faculty of Medicine Hospital between 1 March and 31 May 2026. A control group of adolescents without essential HTN, frequency-matched by age and sex, was also included. The study was conducted in collaboration with the Pediatric Nephrology and Child and Adolescent Psychiatry Departments. Hypertension was confirmed by 24-h ambulatory blood pressure monitoring (ABPM) according to the updated pediatric classification proposed by the American Heart Association (AHA). For participants younger than 13 years, hypertension was defined by a mean 24-h, wake, or sleep systolic or diastolic blood pressure at or above the age-, sex-, and height-specific 95th percentile. For participants aged 13 years or older, ambulatory hypertension was defined by a mean 24-h systolic or diastolic blood pressure ≥125/75 mmHg, a mean wake systolic or diastolic blood pressure ≥130/80 mmHg, or a mean sleep systolic or diastolic blood pressure ≥110/65 mmHg. The ABPM recordings were interpreted according to the 2022 AHA Scientific Statement on ambulatory blood pressure monitoring in adolescents [22]. HTN may involve persistent elevation of systolic blood pressure, diastolic blood pressure, or both at rest and can be categorized as isolated systolic, isolated diastolic, or combined systolic-diastolic HTN [4,22,23]. Twenty-four-hour ambulatory blood pressure monitoring (ABPM) was performed using the Mobil-O-Graph^®^ device (I.E.M. GmbH, Aachen, Germany), an oscillometric ambulatory blood pressure monitor validated according to international standards. An appropriately sized cuff was selected for each child based on midarm circumference with a length of 80–100% of the arm circumference and a width of at least 40% [4]. The daytime (awake) period was defined as 07:00–22:00, and the nighttime (asleep) period as 22:00–07:00. ABPM measurements were obtained at 20-min intervals during the daytime and at 30-min intervals during the nighttime. ABPMs with at least 1 reading per hour, totaling at least 50 readings for a full day, and in which 75% of all possible readings were valid were included in the evaluation. In the control group, office blood pressure was measured using a validated automated oscillometric device with an appropriately sized cuff according to the participant’s arm circumference. Participants were seated quietly for at least 5 min before measurement, with the back supported, feet on the floor, and the arm supported at heart level. Three consecutive office blood pressure measurements were obtained [4,22]. To differentiate essential from secondary HTN and to investigate the underlying etiology, patients underwent a comprehensive evaluation including echocardiography, electrocardiography, detailed urinary tract ultrasonography, renal Doppler ultrasonography, plasma renin and aldosterone levels, blood gas analysis, thyroid function tests, fasting blood glucose, fasting lipid profile, renal and liver function tests, serum electrolytes, urinalysis, fundoscopic examination for hypertensive retinopathy, and assessment of microalbuminuria. Patients diagnosed with HTN were administered the sociodemographic and questionnaire forms before any antihypertensive medication was initiated or lifestyle modifications were recommended.

Hepatic steatosis was assessed by abdominal ultrasonography and graded as grade 1, grade 2, or grade 3 according to the ultrasonographic findings, based on the degree of increased hepatic echogenicity and the visualization of the intrahepatic vessels, diaphragm, and posterior portion of the liver, as previously described by Saverymuttu et al. [24]. All abdominal ultrasonographic examinations were evaluated by experienced radiologists who were blinded to the participants’ behavioral scale results. Ultrasonographic data were available for 43 of the 60 patients with essential hypertension; 17 patients had not undergone abdominal ultrasonography and were therefore excluded from analyses involving hepatic steatosis. Accordingly, these analyses were based on the available data.

Anthropometric measurements were obtained by trained medical personnel in accordance with standardized procedures. Body weight was assessed using a calibrated digital scale and recorded to the nearest 0.1 kg, with participants wearing light clothing. Height was measured to the nearest 0.1 cm using a wall-mounted stadiometer while participants were barefoot. BMI was subsequently calculated by dividing body weight in kilograms by height in meters squared (kg/m^2^). According to age- and sex-specific BMI percentiles, participants were categorized as underweight (<5th percentile), normal weight (5th–< 85th percentile), overweight (85th–< 95th percentile), or obese (≥95th percentile) [25]. These classifications were based on national reference values established for Turkish children [26]. Raw BMI (kg/m^2^) was utilized as the continuous covariate in statistical adjustments.

The control group was selected sequentially using frequency matching based on age and sex criteria (rather than individual one-to-one matching) from among healthy, normotensive children with no history of neurological, nephrological, psychiatric, or other acute or chronic systemic diseases. Both the study and control groups consisted of children who were clinically free of any psychiatric or neurological disorders, as well as any severe sensory or hearing impairments that would prevent meaningful communication. Mental health assessments were conducted clinically. Prior to inclusion in the study, all children underwent a psychiatric evaluation based on DSM-5 criteria by a specialist in child and adolescent psychiatry. All participants were assessed using questionnaire forms regarding grazing, ERD, smartphone addiction, and screen time. The questionnaires were completed by the participants themselves, in the presence of their parents and under the supervision of a child psychiatrist. Lifestyle and sociodemographic variables were assessed using a data collection form developed by the researchers, based on participant and/or parent reports. The variables were assessed based on general/usual behaviors rather than a specific recall period.

### 2.2. Assessment of Grazing Behaviors

Grazing behaviors were assessed using the GQ, developed by Lane and Szabo. The scale is an 8-item, two-factor, 5-point Likert-type (0 = Never, 4 = Always) measure that assesses individuals’ unplanned and repetitive eating of small amounts of food throughout the day, as well as the loss of control experienced during these behaviors. Higher scores indicate more pronounced grazing behaviors [27]. The validity and reliability study of the scale in Turkish was conducted by Akkaya and Yılmaz [28]. Additionally, it has been reported that the scale’s construct validity shows significant correlations with body mass indices, grazing, and emotional schema dimensions [27]. The GQ demonstrated good internal consistency in the present study, with a Cronbach’s alpha coefficient of 0.819.

### 2.3. The Difficulties in Emotion Regulation Scale Short Form (DERS-SF)

DERS-16 scale was used to assess ERD. The scale is a self-report instrument developed as a shorter version of the original 36-item scale (DERS-SF). It consists of a total of 16 items, and each item is rated on a 5-point scale (e.g., ranging from “Almost never” to “Almost always”). A higher total score indicates more pronounced difficulties in emotion regulation [29]. The Turkish version of the scale was validated and its reliability was established by Demirpence et al. [30]. In the present study, the DERS-SF showed high internal consistency, with a Cronbach’s alpha coefficient of 0.920.

### 2.4. Smartphone Addiction Scale—Short Version (SAS-SV)

The Smartphone Addiction Scale—Short Version (SAS-SV) was used to assess problematic smartphone use among participants. The SAS-SV was developed and validated specifically for adolescents by Kwon et al. (2013) [12] and consists of 10 items rated on a 6-point Likert scale, ranging from 1 (strongly disagree) to 6 (strongly agree). Total scores range from 10 to 60, with higher scores indicating greater levels of problematic smartphone use [12]. The Turkish version of the SAS-SV was adapted and validated for adolescents by Şata and Karip, demonstrating satisfactory validity and reliability [31]. In the present study, the Cronbach’s alpha coefficient for the SAS-SV was 0.845.

### 2.5. Statistical Analysis

Sample size estimation was determined prior to data collection using GPower software (version 3.1.9.4). Based on the prior literature investigating grazing and atypical eating behaviors in pediatric cohorts (Conceição et al., 2021) [32], a medium effect size (Cohen’s d = 0.50 to 0.57, corresponding to Cohen’s f ≈ 0.25–0.28) was anticipated for primary outcome comparisons between clinical and non-clinical groups. Assuming an independent two-sample comparison/ANCOVA framework at α = 0.05 and 80% statistical power, a minimum sample size of 100 participants (50 per group) was required. Our final enrolled sample of *n* = 120 (60 per group) exceeded this threshold, ensuring adequate statistical power (>80%) for evaluating group differences on the primary GQ outcome. Secondary subgroup analyses involving hepatic steatosis (*n* = 43) were recognized as having restricted statistical power and were interpreted strictly as exploratory. Statistical analyses were performed using IBM SPSS Statistics for Windows, Version 23.0 (IBM Corp. Released 2017. IBM SPSS Statistics for Windows, Version 23.0. Armonk, NY, USA: IBM Corp.). Descriptive analyses included the assessment of participants’ age and sex distributions, as well as the frequencies and percentages of the clinical characteristics of the study population. Continuous variables were reported as mean ± standard deviation and median (minimum–maximum). The distributional characteristics of continuous variables were evaluated based on skewness and kurtosis values, with values ranging from −1.5 to +1.5 considered indicative of an approximately normal distribution. For comparisons between two groups, the independent-samples *t*-test was applied to continuous variables meeting the normality assumption, whereas the Mann–Whitney U test was used for variables that did not satisfy this assumption. For comparisons involving three groups, one-way analysis of variance (ANOVA) was performed when the normality assumption was fulfilled, while the Kruskal–Wallis test was applied when the assumption was not met. Because group recruitment was based on frequency matching rather than individual paired matching, standard independent two-sample statistical tests (e.g., Independent *t*-test, Mann–Whitney U test, and Chi-square test) and Analysis of Covariance (ANCOVA) were appropriately applied for group comparisons. Prior to executing the Analysis of Covariance (ANCOVA), all underlying statistical assumptions were systematically evaluated. The linearity between the covariate (BMI) and the dependent variables was confirmed through scatterplot inspections. The assumption of homogeneity of regression slopes was formally tested by evaluating the interaction terms between group and BMI (Group × BMI) for each outcome variable, none of which reached statistical significance (*p* > 0.05). Levene’s test indicated that the variances were homogeneous (*p* > 0.05). The normality and homoscedasticity of the residuals were subsequently evaluated using the Shapiro–Wilk test and Q-Q plots, respectively, and both assumptions were satisfied. Finally, adequate distributional overlap of BMI between groups was visually confirmed prior to model execution. Raw BMI (kg/m^2^) was included as the continuous covariate in Analysis of Covariance (ANCOVA) models to adjust for group differences in body weight. To evaluate the primary study hypotheses while statistically controlling for potential confounding by body mass index (BMI), Analysis of Covariance (ANCOVA) models were constructed as the principal inferential analyses. ANCOVA was specifically selected to evaluate BMI-adjusted between-group differences on clinical outcomes. In the first set of ANCOVA models, Group status (HTN vs. Healthy Control) served as the two-level fixed factor, while total and subscale scores of the Grazing Questionnaire (GQ), DERS-16, and SAS were specified as separate continuous dependent variables. In secondary ANCOVA models, the presence of hepatic steatosis (Yes vs. No) within the HTN group served as the fixed factor. In all models, BMI was entered as the primary continuous covariate. Prior to model execution, core ANCOVA assumptions were formally evaluated: linearity was verified via scatterplots, homogeneity of regression slopes was confirmed by non-significant Group × Covariate interaction terms (*p* > 0.05), error variance homogeneity was evaluated via Levene’s test, and residual normality was confirmed using diagnostic plots. Partial eta squared (ηp^2^) was used to quantify effect sizes, with 0.01, 0.06, and 0.14 corresponding to small, moderate, and large effect sizes, respectively. Missing data (e.g., incomplete hepatic steatosis evaluations) were handled using listwise deletion. The total GQ score was treated as the primary outcome in the present analysis, whereas all other endpoints—including GQ subscales (Grazing Behavior and Uncontrollability), secondary scales (DERS-16, SAS), and subgroup analyses based on hepatic steatosis—were considered exploratory secondary outcomes. To prevent unnecessary inflation of Type II error in exploratory end points, adjustments for multiple testing (e.g., Bonferroni or False Discovery Rate procedures) were not performed. Consequently, findings with *p*-values close to the 0.05 threshold (particularly secondary psychosocial correlations and hepatic steatosis subgroup comparisons) should be interpreted as hypothesis-generating, and the associated potential for Type I error is acknowledged. Statistical inferences were guided jointly by *p*-values, effect sizes (ηp^2^), and 95% Confidence Intervals (95% CIs). Associations among the clinical scale scores were assessed using Pearson’s correlation coefficient for normally distributed variables and Spearman’s correlation coefficient for variables that deviated from a normal distribution. The level of statistical significance for all hypotheses was accepted as *p* < 0.05.

## 3. Results

### 3.1. Demographic and Clinical Characteristics of Participants

No significant differences were found between the groups in terms of age and sex (*p* > 0.05). In the HTN group, 40 (66.7%) participants were male, while in the control group, 33 (55.0%) participants were male (*p* = 0.190).

When the participants’ anthropometric and clinical characteristics were examined, the mean BMI in the HTN group was found to be statistically significantly higher (31.18 ± 6.81 kg/m^2^) than that of the control group (23.35 ± 4.47 kg/m^2^) (*p* < 0.001). When BMI was classified categorically, it was found that a significant percentage of individuals in the HTN group (81.7%, *n* = 49) were overweight or obese, whereas this percentage remained at 28.3% (*n* = 17) in the control group (*p* < 0.001) (Table 1).

When the presenting complaints of patients at admission in the HTN group were evaluated, it was determined that 56.7% (*n* = 34) of them received an incidental diagnosis during routine checkups, followed by headache in 30.0% (*n* = 18), chest pain in 6.7% (*n* = 4), and palpitations in 6.7% (*n* = 4) of the patients. Additionally, patients had both systolic and diastolic HTN (*n* = 31:51.7%), isolated systolic HTN (*n* = 21:35.0%), and isolated diastolic HTN (*n* = 8:13.3%). On the other hand, no statistically significant difference was found between the HTN group (7.60 ± 1.05 h/day) and the control group (7.76 ± 0.85 h/day) in terms of average daily sleep duration (*p* = 0.367). Furthermore, no significant differences were found between the groups in terms of weekly physical activity levels and frequency of sugary beverage and fast food consumption (Table 1). However, a statistically significant difference was observed between the groups regarding the pattern of eating in front of the screen (*p* = 0.030), with a higher proportion of healthy controls reporting occasional screen-associated eating (60.0% vs. 35.0%), whereas frequent or always eating in front of the screen was reported by 26.6% of the essential HTN group and 20.0% of the control group (Table 1).

### 3.2. Relationships Among Clinical Variables in the Entire Sample

Pearson correlation analyses revealed that total GQ scores were positively correlated with BMI (r = 0.252, *p* = 0.006), difficulties (r = 0.187, *p* = 0.041), and smartphone addiction (r = 0.315, *p* < 0.001). While high correlations were observed between the GQ total score and its constituent subscales (Grazing Behaviors: r = 0.926; Uncontrollability: r = 0.844), these expected part-whole correlations represent mathematical dependencies inherent to scale structure rather than independent clinical relationships. Importantly, ERD was significantly correlated with the Uncontrollability subscale (r = 0.248, *p* = 0.006) rather than overall grazing behaviors (Table 2).

### 3.3. Comparison of Clinical Scale Scores of the HTN and Control Groups

An Analysis of Covariance (ANCOVA) was performed to compare the levels of clinical symptoms between the HTN and control groups. To statistically control for the potential confounding effect of the observed difference in BMI between the groups on the dependent variables, BMI was included in the model as a covariate (control variable).

The ANCOVA model established to predict grazing behavior was found to be statistically significant as a whole [Model F(2, 117) = 5.72, *p* = 0.004, η^2^p = 0.089, adjusted R^2^ = 0.073]. It was found that BMI had no statistically significant effect on grazing behavior (*p* = 0.179, η^2^p = 0.015). No statistically significant difference was found between the HTN and the control groups in terms of the grazing behavior subscale scores (*p* = 0.075, η^2^p = 0.027). The ANCOVA model established to estimate Uncontrollability scores was found to be statistically significant as a whole [F(2, 117) = 4.451, *p* = 0.014, η^2^p = 0.071, adjusted R^2^ = 0.055] (Table 3). It was determined that BMI had no statistically significant effect on uncontrollability scores, but the difference in uncontrollability scores between the HTN and the control groups was statistically significant [F(1, 117) = 4.64, *p* = 0.033, η^2^p = 0.038]. Higher levels of uncontrollability were observed in the HTN group (Table 3).

The model developed to predict the total scores of the Grazing Scale was found to yield statistically significant results [Model F(2, 117) = 6.54, *p* = 0.002, η^2^p = 0.101, adjusted R^2^ = 0.085]. The difference between the HTN group and the control group in terms of total scores of the Grazing Scale was found to be statistically significant [F(1, 117) = 4.85, *p* = 0.030, η^2^p = 0.040]. This finding indicates that, after adjustment for BMI, adolescents with essential HTN had significantly higher total GQ scores than control group (Table 3).

The ANCOVA model established to detect differences in levels of ERD (*p* = 0.468) and smartphone addiction (*p* = 0.613) was found to be non-statistically significant as a whole (Table 3).

### 3.4. Comparison of Clinical Symptoms Between Individuals with and Without Hepatic Steatosis in the HTN Group

To ensure that the missing ultrasound records for 17 participants in the HTN group did not introduce selection bias, subgroup comparisons were performed between hypertensive patients with (*n* = 43) and without (*n* = 17) available liver imaging. No statistically significant differences were found between these two subgroups regarding age (*p* = 0.921), sex distribution (*p* = 0.375), baseline BMI (*p* = 0.305), or GQ total scores (*p* = 0.478). While these findings confirm that the two subgroups were comparable across the evaluated baseline parameters, the exact mechanism underlying the missing liver-imaging data cannot be definitively established.

The results of the BMI-adjusted ANCOVA, conducted to examine the impact of the presence of hepatic steatosis on scale scores in the essential HTN group, are presented in Table 4. The results of the analysis revealed that BMI, when included in the model as a control variable, had no significant main effect on any of the scale scores (*p* > 0.05). Even when participants’ BMI levels were controlled for, patients with hepatic steatosis had significantly higher total scores on their GQ scores (*p* = 0.019, η^2^p = 0.138), “Grazing Behaviors” subscale (*p* = 0.046, η^2^p = 0.107), and their “Uncontrollability” subscale scores (*p* = 0.025, η^2^p = 0.123) when compared with those of patients without hepatic steatosis. However, no statistically significant difference was found between both groups in terms of levels of ERD (*p* = 0.454) and smartphone addiction (*p* = 0.262). The grazing behavior, Uncontrollability, and GQ scores of hypertensive patients with hepatic steatosis (adjusted mean ± SE: 11.17 ± 0.99; 5.57 ± 0.89, and 16.75 ± 1.68) were found to be significantly higher compared to patients without hepatic steatosis (adjusted mean ± SE: 8.31 ± 0.81; 2.66 ± 0.73; 10.97 ± 1.39) (Table 4).

## 4. Discussion

Our findings suggest that adolescents with essential HTN have higher grazing behavior scores than adolescents without essential HTN after adjustment for BMI. A significant positive Pearson correlation was observed between total grazing scores and BMI, ERD and smartphone addiction. In line with subdimension-specific patterns, ERD was specifically linked with the sense of uncontrollability during eating, whereas high correlations between total grazing scores and its subscales were interpreted as structural part–whole dependencies rather than distinct behavioral associations. To the best of our knowledge, this is the first study to specifically evaluate grazing behavior in adolescents with essential HTN characterized by 24-h ABPM while also examining its associations with obesity, ERD, problematic smartphone use, and screen time. After adjustment for BMI, adolescents with essential HTN had significantly higher total GQ scores, particularly in the Uncontrollability dimension, than adolescents without essential HTN. These findings indicate an association between grazing behavior and essential HTN, although the cross-sectional nature of the study precludes establishing causal or temporal associations. This finding indicates that focusing solely on metabolic risk factors may not be sufficient in the assessment of essential HTN in adolescence and highlights the importance of including eating behavior characteristics in the clinical evaluation. Adolescence represents a critical period during which lifestyle and health-related behaviors undergo substantial changes [33]. Therefore, identifying factors that influence eating behavior during childhood and adolescence may contribute to the early recognition and prevention of unhealthy eating behaviors. Studies have shown that loss-of-control eating in children and adolescents is associated with overweight and obesity and may increase the risk of subsequent weight gain [34]. In addition, children experiencing loss-of-control eating episodes have been reported to consume a higher proportion of calories from snacks and desserts [35]. In a study by Conceição et al., higher levels of grazing behavior in children were associated with anxiety and depressive symptoms, body image dissatisfaction, and inappropriate eating habits [32]. Furthermore, studies have shown that children who experience eating episodes characterized by a loss of control have weaker emotion regulation skills [36,37]. Previous research has reported associations between excessive internet use, internet addiction, and adverse physical and psychological health outcomes among adolescents [15]. In our study, grazing behaviors were associated with ERD and problematic smartphone use. The association between grazing behavior and problematic smartphone use may suggest a potential link between problematic digital-device use and problematic eating behaviors. In our study, grazing behaviors were associated with BMI, ERD, and problematic smartphone use. The role of BMI in this association warrants careful consideration, as BMI may function either as a potential confounding factor or as an intermediate factor in the relationship between grazing behavior and essential HTN. Therefore, adjustment for BMI may have attenuated part of the association under investigation. In particular, the positive association between grazing behavior and smartphone addiction supports potential behavioral links between screen exposure, irregular eating patterns, and energy intake control in modern lifestyles. However, ERD and problematic smartphone use did not differ significantly between the HTN and control groups, although both were associated with grazing behavior across the study sample.

In exploratory subgroup analyses, the presence of hepatic steatosis within the HTN group exhibited preliminary trends toward higher grazing scores and a greater sense of uncontrollability over eating. In BMI-adjusted analyses, hypertensive individuals with hepatic steatosis exhibited higher levels of grazing behavior, a greater sense of uncontrollability, and higher total grazing scores. While these BMI-adjusted associations suggest a potential link between eating behaviors and hepatic steatosis, the substantial overlap between obesity and hepatic steatosis requires cautious interpretation regarding the independent role of adiposity. It has been observed that not only excess body weight but also dietary quality plays a critical role in the development of hepatic steatosis associated with childhood obesity. In particular, diets high in simple carbohydrates, especially fructose, and saturated fats may promote the development of hepatic steatosis, whereas dietary fiber and healthier dietary patterns may have beneficial effects [38,39]. For this reason, current pediatric guidelines recommend lifestyle modification, including improvement of dietary quality and increased physical activity, as the first-line approach to the management of pediatric hepatic steatosis. Hepatic steatosis in children and adolescents has been associated with unhealthy dietary habits, including less frequent breakfast consumption and more frequent intake of fast food and snacks [40]. For this reason, current pediatric guidelines recommend lifestyle modification, including improvement of dietary quality and increased physical activity, as the first-line approach to the management of pediatric hepatic steatosis [41]. In addition, reducing the intake of free sugars and sugar-sweetened foods and beverages may improve hepatic steatosis in pediatric patients with NAFLD [42]. Current recommendations suggest 9–11 h of sleep for children aged 5–13 years and 8–10 h for adolescents aged 14–17 years [43]. In our study, no statistically significant difference was found between the HTN group (7.60 ± 1.05 h/day) and the control group (7.76 ± 0.85 h/day) in terms of average daily duration of sleep (*p* = 0.367). Previous research has shown that insufficient sleep duration is associated with unhealthy dietary habits, increased screen time, and overweight/obesity in children [44]. No statistically significant between-group differences were detected in average sleep duration or screen time, as well as in weekly physical activity levels, frequency of sugary drink consumption, or fast-food intake. The findings suggest a potential association between grazing behavior and essential HTN, as well as metabolic indicators such as hepatic steatosis. However, given the exploratory nature and small sample size of the hepatic steatosis subgroup analysis, these findings should be interpreted cautiously as hypothesis-generating preliminary observations.

### Study Limitations

This study has several limitations. First, due to its cross-sectional design, the causality of the relationships between grazing behavior, essential HTN, and hepatic steatosis cannot be assessed. Second, the single-center setting and relatively limited sample size may restrict the generalizability of the study findings. Additionally, grazing behaviors, ERD, and smartphone addiction were assessed using self-report scales, which may have led to response bias. Thirdly, while statistical adjustments were made for BMI, baseline differences between groups in family history of HTN and obesity were not included in the multivariable models due to sample size constraints. Consequently, residual confounding from unmeasured genetic, environmental, or familial cardiometabolic factors cannot be entirely ruled out. Finally, variables such as ERD and problematic smartphone use were evaluated in bivariate and secondary analyses rather than adjusted for simultaneously within a comprehensive multivariable framework; thus, independence from these psychosocial constructs should not be inferred. Additionally, due to the exploratory nature of secondary scale evaluations and subgroup analyses involving multiple statistical comparisons, formal adjustments for multiplicity (e.g., Bonferroni correction) were not applied. Consequently, *p*-values close to the conventional threshold (*p* = 0.02–0.05) should be interpreted cautiously as hypothesis-generating rather than definitive evidence of association. Due to the exploratory nature of several secondary end points and subgroup evaluations, adjustments for multiple testing were not executed. Given that several observed *p*-values approached the conventional 0.05 threshold, the possibility of inflated Type I error cannot be excluded, and these secondary findings require confirmation in larger, prospectively powered cohorts. Additionally, hepatic ultrasonography data were unavailable for 17 of the 60 hypertensive participants (28.3%), resulting in a reduced sample size for this subgroup analysis (*n* = 43) and lower statistical power (~44.6%). Although comparative analyses between participants with and without available liver imaging revealed no significant differences in baseline parameters, the exact mechanism underlying the missing data cannot be definitively established, and potential selection bias cannot be completely ruled out. Therefore, findings regarding hepatic steatosis should be viewed with caution as exploratory and hypothesis-generating. Finally, although BMI was included as a covariate in ANCOVA models, the substantial baseline difference in BMI between the HTN and control groups represents a notable limitation. Because the BMI distributions of the two groups overlap only partially, the statistical adjustment may not fully eliminate potential confounding by adiposity. Finally, raw BMI (kg/m^2^) rather than age- and sex-standardized BMI z-scores (BMI SDS) was used as the covariate in statistical adjustments due to data collection constraints. Although frequency matching for age and sex minimized age- and sex-dependent growth variance between groups, future prospective studies utilizing standardized BMI SDS measures are recommended. On the other hand, the limited psychometric evidence for the Turkish version of the GQ, specifically in adolescents aged 12–18 years, should be considered when interpreting the findings. Masked HTN could not be definitively excluded in the control group because ABPM was not performed in participants with normal office blood pressure measurements.

## 5. Conclusions

Adolescents with essential HTN had higher BMI-adjusted GQ scores, particularly in the Uncontrollability dimension, than adolescents without essential HTN. GQ scores were also associated with BMI, ERD, and problematic smartphone use across the study sample. Given the substantial overlap between essential HTN and elevated body weight, these findings should be interpreted cautiously. Nevertheless, grazing behavior may be a relevant behavioral characteristic to consider in the clinical assessment of adolescents with essential HTN. In adolescents with essential HTN, grazing behavior, obesity, problematic smartphone use, and screen exposure warrant multidisciplinary monitoring by pediatric healthcare professionals, including child and adolescent psychiatrists. It is essential to adopt a healthy lifestyle starting in childhood and to raise public awareness on this issue. Multicenter, prospective and longitudinal studies are needed to clarify the temporal directionality and potential causal relationships among grazing behavior, adiposity, and essential HTN and to evaluate the effects of behavioral interventions on cardiometabolic outcomes.

## Figures and Tables

**Table 1:** Comparison between the patient and the control group in terms of demographic and clinical characteristics.

Characteristic Features		Patient Group	Control Group	*p*
Age, mean ± SD		15.02 ± 1.98	15.14 ± 1.77	0.710 ^a^
Gender (male/female) *n* (%)		40 (66.7)/20 (33.3)	33 (55.0)/27 (45.0)	0.190 ^b^
Maternal age, years, mean ± SD		40.98 ± 5.16	41.85 ± 4.37	0.323 ^a^
Paternal age, years, mean ± SD		45.76 ± 5.74	45.66 ± 4.42	0.912 ^a^
Educational level of the mother, *n* (%)	Primary school	20 (33.30)	23 (38.3)	0.174 ^b^
	Middle school	20 (33.30)	10 (16.7)	
	High school	10 (16.7)	16 (26.7)	
	University	10 (16.7)	11 (18.3)	
Educational level of the father. *n* (%)	Primary school	13 (21.7)	13 (21.7)	0.978 ^b^
	Middle school	10 (16.7)	11 (18.3)	
	High school	20 (33.3)	21 (35.0)	
	University	17 (28.3)	15 (25.0)	
Mother is employed/unemployed, *n* (%)		14 (23.3)/46 (76.7)	13 (21.7)/47 (78.3)	0.827 ^b^
Father is employed/unemployed, *n* (%)		53 (88.3)/7 (11.7)	55 (91.7)/5 (8.3)	0.543 ^b^
Body weight (kg), mean ± SD		88.75 ± 26.50	64.44 ± 17.87	<0.001 ^a^
Height (cm), mean ± SD		166.86 ± 12.02	164. 75 ± 10.54	0.307 ^a^
BMI (kg/m^2^), mean ± SD		31.18 ± 6.81	23.35 ± 4.47	<0.001 ^a^
According to BMI percentiles, *n* (%)	Thin and Normal weight	11 (18.3)	43 (71.7)	<0.001 ^c^
	Overweight and obese	49 (81.7)	17 (28.3)	
Family history of HTN, (yes/no) *n*/%		23 (38.3)/37 (61.7)	10 (16.7)/50 (83.3)	0.008 ^b^
Family history of obesity, (yes/no) *n*/%		15 (25.0)/45 (75.0)	6 (10.0)/54 (90)	0.031 ^b^
Family history of diabetes, (yes/no) *n*/%		3 (5.0)/57 (95.0)	2 (3.3)/58 (96.7)	0.648 ^c^
Family history of dyslipidemia, (yes/no) *n*/%		1 (1.7)/59 (98.3)	0/(0.0)/(60)	1.000 ^c^
Family history of psychiatric illness (yes/no) *n*/%		3 5.0)/57 (95.0)	3(5.0)/57 (95.0)	1.000 ^c^
Complaints of the patients, *n* (%)	Incidental	34 (56.7)		
	Headache	18 (30.0)		
	Chest pain	4 (6.7)		
	Palpitations	4 (6.7)		
Hepatic steatosis (yes/no) *		18/25		
Type of HTN, *n* (%)	Systolic	21 (35.0)		
	Diastolic	8 (13.3)		
	Both systolic and diastolic	31 (51.7)		
Treatments received, *n* (%)	ACEi	16 (26.7)		
	Ca channel blockers	38 (63.3)		
	Multidrug therapy	6 (10.0)		
Secondhand exposure to cigarette smoke (yes/no), *n* (%)		21(35.0)/39 (65.0)	22(36.7)/38 (63.3)	0.849 ^b^
Averagedaily duration of sleep (hours), mean ± SD		7.60 ± 1.05	7.76 ± 0.85	0.367 ^a^
Sleeping problems (yes/no), *n* (%)		10 (16.7)/50 (83.3)	5 (10.0)/55 (90.0)	0.168 ^b^
Physical activity per day (days/week) mean ± SD		1.31 ± 1.03	1.63 ± 1.11	0.271 ^a^
Consumption of sugary drinks, *n* (%)	Rarely	13 (21.7)	22 (36.6)	0.334 ^b^
	1–2 days a week	24 (40.0)	18 (30.0)	
	3–4 days a week	11 (18.3)	10 (16.7)	
	5–7 days a week	12 (20.0)	10 (16.7)	
Consumption of fast food, *n* (%)	Rarely	23 (38.3)	21 (35.0)	0.672 ^b^
	Monthly	15 (25.0)	19 (31.7)	
	Once a week	10 (16.7)	12 (20.0)	
	More than once a week	12 (20.0)	8(13.3%)	
Weekday screen time (hours/day) mean ± SD		1.98 ± 0.92	1.76 ± 0.92	0.204 ^a^
Weekend screen time (hours/day) mean ± SD		2.35 ± 0.91	2.25 ± 0.83	0.534 ^a^
The most frequently used media device, *n* (%)	Phone	47 (78.4)	48 (80.0)	0.616 ^b^
	Tablet computer	3 (5.0)	5 (8.3)	
	Computer	5 (8.3)	5 (8.3)	
	Tv	5 (8.3)	2 (3.4)	
Screen usebefore falling asleep, *n* (%)		46 (76.7)/14 (23.3)	40 (66.7)/20 (33.3)	0.224 ^b^
Falling asleep in front of screen, *n* (%)		22 (36.7)/38 (63.3)	16 (26.7)/44 (73.3)	0.239 ^b^
Parental control (yes/no), *n* (%)		36 (60.0)/24 (40.0)	36 (60.0)/24 (40.0)	1.000 ^b^
Eating in front of the screen (yes/no), *n* (%)	Never	23 (38.3)	12 (20.0)	0.030 ^b^
	Occasionally	21 (35.0)	36 (60.0)	
	Frequently	5 (8.3)	6 (10.0)	
	Always	11 (18.3)	6 (10.0)	

Footnote: Antihypertensive treatments (ACEi, Ca channel blockers, multidrug therapy) listed for the essential HTN group were initiated after the baseline questionnaire assessments and diagnostic evaluations were completed. Abbreviations: BMI: Body Mass Index. * data of 17 cases are missing. a: Independent *t*-test, b: chi-square test, c: Fisher’s Exact Test.

**Table 2:** Relationships among clinical variables in the entire sample.

		BMI	Grazing Behaviors	Uncontrollability	GQ-Total	DERS-16	SAS-SV
**BMI**	*r*	1.000					
	*p*	-					
**Grazing Behaviors**	*r*	0.253	1.000				
	*p*	0.005	-				
**Uncontrollability**	*r*	0.184	0.579	1.000			
	*p*	0.044	<0.001	-			
**GQ-total**	*r*	0.252	0.926	0.844	1.000		
	*p*	0.006	<0.001	<0.001	-		
**DERS-16**	*r*	0.098	0.110	0.248	0.187	1.000	
	*p*	0.289	0.230	0.006	0.041	-	
**SAS-SV**	*r*	−0.031	0.270	0.295	0.315	0.334	1.000
	*p*	0.737	0.003	0.001	<0.001	<0.001	-

*r* = Pearson correlation coefficient. Abbreviations: BMI: Body Mass Index; GQ: Grazing Questionnaire; DERS-16: Difficulties in Emotion Regulation Scale—Brief Form; SAS-SV: Smartphone Addiction Scale—Short Version.

**Table 3:** Comparison Of Clinical Scales Across Groups.

Variable	HT Group (*n* = 60) Adjusted Mean ± SE [95% CI]	Control Group (*n* = 60) Adjusted Mean ± SE [95% CI]	Covariate (BMI) F (1, 117) *p* [ηp^2^]	Main Effect (Group) F (1, 117) *p* [ηp^2^]	Corrected Model F (2, 117) *p* [ηp^2^]
**Grazing Behaviors**	9.38 ± 0.57 [8.26–10.50]	7.80 ± 0.57 [6.68–8.92]	1.826 *p* = 0.179 [0.015]	3.219 *p* = 0.075 [0.027]	5.720 ***p* = 0.004** 0.089
**Uncontrollability**	3.814 ± 0.405 [3.02–4.61]	2.469 ± 0.405 [1.68–3.26]	0.235 *p* = 0.629 [0.002]	4.641 ***p* = 0.033** [0.038]	4.451 ***p* = 0.014** 0.071
**GQ-total**	13.19 ± 0.861 [11.50–14.88]	10.27 ± 0.861 [8.58–11.96]	1.261 *p* = 0.264 [0.011]	4.847 ***p* = 0.030** [0.040]	6.542 ***p* = 0.002** **0.101**
**DERS-16**	31.92 ± 1.816 [28.36–35.48]	33.694 ± 1.816 [30.13–37.25]	1.524 *p* = 0.219 [0.013]	0.400 *p* = 0.528 [0.003]	0.765 *p* = 0.468 0.013
**SAS-SV**	25.59 ± 1.392 [22.86–28.32]	27.602 ± 1.392 [24.87–30.33]	0.062 *p* = 0.803 [0.001]	0.870 *p* = 0.353 [0.007]	0.492 *p* = 0.613 0.008

Covariates appearing in the model are evaluated at the following values: BMI = 27.2666. Abbreviations: GQ: Grazing Questionnaire; DERS-16 Difficulties in Emotion Regulation Scale—Brief Form; SAS-SV: Smartphone Addiction Scale—Short Version.

**Table 4:** Comparison of clinical symptoms between individuals with and without hepatic steatosis in the HTN group.

Variable	Hepatic Steatosis (+) (*n* = 18) Adjusted Mean ± SE [95% CI]	Hepatic Steatosis (−) (*n* = 25) Adjusted Mean ± SE [95% CI]	Covariate (BMI) F (1, 40) *p* [ηp^2^]	Main Effect (Group) F (1, 40) *p* [ηp^2^]	Corrected Model F (2, 40) *p* [ηp^2^]
**Grazing Behaviors**	11.17 ± 0.99 [9.23–13.11]	8.31 ± 0.81 [6.72–9.90]	0.277 *p* = 0.601 [0.007]	4.262 *p* = 0.046 [0.096]	2.395 *p* = 0.104 0.107
**Uncontrollability**	5.57 ± 0.89 [3.83–7.31]	2.66 ± 0.73 [1.23–4.09]	0.790 *p* = 0.379 [0.019]	5.400 *p* = 0.025 [0.119]	2.808 *p* = 0.072 0.123
**GQ-total**	16.75 ± 1.68 [13.46–20.04]	10.97 ± 1.39 [8.25–13.69]	0.607 *p* = 0.440 [0.015]	5.957 *p* = 0.019 [0.130]	3.204 *p* = 0.051 0.138
**DERS-16**	35.08 ± 3.60 [28.02–42.14]	31.25 ± 2.97 [25.43–37.07]	0.156 *p* = 0.695 [0.004]	0.572 *p* = 0.454 [0.014]	0.756 *p* = 0.476 0.036
**SAS-SV**	27.69 ± 2.56 [22.67–32.71]	23.58 ± 2.11 [19.44–27.72]	0.297 *p* = 0.589 [0.007]	1.292 *p* = 0.262 [0.031]	0.651 *p* = 0.527 0.032

Covariates of the model are evaluated at the following values: BMI = 31.8014. Abbreviations: BMI: Body Mass Index; GQ: Grazing Questionnaire; DERS-16: Difficulties in Emotion Regulation Scale–Brief Form; SAS-SV: Smartphone Addiction Scale–Short Version.

## Data Availability

The datasets generated and analyzed during the current study are available from the corresponding author upon reasonable request. They are not publicly accessible because of privacy, ethical, and confidentiality requirements related to participants’ health information.
